# An unexpectedly long history of sexual selection in birds-of-paradise

**DOI:** 10.1186/1471-2148-9-235

**Published:** 2009-09-16

**Authors:** Martin Irestedt, Knud A Jønsson, Jon Fjeldså, Les Christidis, Per GP Ericson

**Affiliations:** 1 Molecular Systematics Laboratory, Swedish Museum of Natural History, P.O. Box 50007, SE-104 05 Stockholm, Sweden; 2 Vertebrate Department, Zoological Museum, University of Copenhagen, Universitetsparken 15, DK-2100 Copenhagen Ø, Denmark; 3 Division of Research and Collections, Australian Museum, 6 College St, Sydney, New South Wales 2010, Australia; 4 Department of Genetics, University of Melbourne, Parkville, Victoria 3052, Australia

## Abstract

**Background:**

The birds-of-paradise (Paradisaeidae) form one of the most prominent avian examples of sexual selection and show a complex biogeographical distribution. The family has accordingly been used as a case-study in several significant evolutionary and biogeographical syntheses. As a robust phylogeny of the birds-of-paradise has been lacking, these hypotheses have been tentative and difficult to assess. Here we present a well supported species phylogeny with divergence time estimates of the birds-of-paradise. We use this to assess if the rates of the evolution of sexually selected traits and speciation have been excessively high within the birds-of-paradise, as well as to re-interpret biogeographical patterns in the group.

**Results:**

The phylogenetic results confirm some traditionally recognized relationships but also suggest novel ones. Furthermore, we find that species pairs are geographically more closely linked than previously assumed. The divergence time estimates suggest that speciation within the birds-of-paradise mainly took place during the Miocene and the Pliocene, and that several polygynous and morphologically homogeneous genera are several million years old. Diversification rates further suggest that the speciation rate within birds-of-paradise is comparable to that of the enitre core Corvoidea.

**Conclusion:**

The estimated ages of morphologically homogeneous and polygynous genera within the birds-of-paradise suggest that there is no need to postulate a particularly rapid evolution of sexually selected morphological traits. The calculated divergence rates further suggest that the speciation rate in birds-of-paradise has not been excessively high. Thus the idea that sexual selection could generate high speciation rates and rapid changes in sexual ornamentations is not supported by our birds-of-paradise data. Potentially, hybridization and long generation times in polygynous male birds-of-paradise have constrained morphological diversification and speciation, but external ecological factors on New Guinea may also have allowed the birds-of-paradise to develop and maintain magnificent male plumages. We further propose that the restricted but geographically complex distributions of birds-of-paradise species may be a consequence of the promiscuous breeding system.

## Background

Birds-of-paradise (Paradisaeidae) are renowned for their complex courtships and diverse male plumages with highly elongated and elaborate feathers. Arguably, the birds-of-paradise form one of the most extravagant clades of all bird families in terms of beauty and display behaviour. The degree of sexual dimorphism found in the majority of species of birds-of-paradise (cryptically coloured females while males have spectacular ornamented plumages and elaborate courtship displays) is one of the most prominent avian examples of sexual selection. The birds-of-paradise also show complex biogeographical patterns, where most species and subspecies are locally and disjunctly distributed, sometimes in seemingly uniform and interconnected landscapes (summarized in [1]). Consequently, the family has been used as one of the earliest case-studies in significant evolutionary and biogeographical syntheses (e.g., [2-4]). Despite the evolutionary interest in the family, no comprehensive robust phylogeny of the birds-of-paradise exists.

Several incongruent phylogenetic hypotheses have been proposed for the birds-of-paradise based on both morphology [5-8] and molecular distances [9-11]. The only molecular phylogenetic study [12] lacks several genera and is based only on a single mitochondrial marker. Recent molecular studies have demonstrated that some taxa, traditionally considered to belong to the birds-of-paradise, are more closely related to other passerine clades. Thus, Macgregor's bird-of-paradise (*Macgregoria pulchra*) is a giant honeyeater [13], while the cnemophiline birds-of-paradise form a distinct lineage well separated from the true birds-of-paradise clade [13-15]. The lesser melampitta (*Melampitta lugubris*) and the silktail (*Lamprolia victoriae*) have also been proposed to be related to the birds-of-paradise [10,16,17], but are now confidently placed outside this clade [14,15,18].

The Fisherian runaway model of sexual selection [19,20] suggests that sexual selection could generate a rapid and continual change in sexual ornaments, as few males get most of the mating and are free from constraints imposed by parental behaviour. In lekking species, sexual selection and dimorphism are expected to be even stronger (e.g., [3,21]. The power of sexual selection to drive changes in mate recognition traits is also potentially a potent force in speciation (e.g., [22-25]). However, some models suggest that strong sexual selection may act against speciation [26].

Here we present a robust phylogenetic hypothesis and divergence time estimates for the birds-of-paradise (*stricto sensu*), based on both nuclear and mitochondrial sequence data. We use this i) to asses if the speciation rate and the evolution of sexually selected traits have been excessively high in the polygynous core birds-of-paradise, and ii) to examine biogeographical patterns and analyze the geographical differentiation in relation to sexually selected traits.

## Methods

### Taxon sampling, amplification and sequencing

We examined the phylogenetic relationship among birds-of-paradise by analyzing DNA sequence data from the mitochondrial cytochrome *b *gene and the two nuclear loci ornithine decarboxylase introns 6 to 7 (ODC), and glyceraldehyde-3-phosphodehydrogenase intron 11 (GAPDH). The taxon sampling includes all 40 species of birds-of-paradise recognized by Monroe and Sibley [27], omitting the genera *Loboparadisea*, *Cnemophilus*, *Melampitta *and *Macgregoria*, which have been shown to not be part of the birds-of-paradise clade [13-15]. The taxon sampling also includes *Corvus cornix *and *Monarcha melanopsis*, two taxa representing lineages that have been suggested to be closely related to the birds-of-paradise [14]. Three additional but more distantly related representatives of core Corvoidea as well as representatives from other major passerine lineages were also included. A representative from Psittacidae, the sister group to passerine birds, was used to root the trees. See Table 1 for the taxon sampling and GenBank accession numbers.

**Table 1:** Specimen data and Genbank accession numbers for samples used in the study.

**Vernacular name**	***Scientific name***	**Sample id**.	**G3P**	**ODC**	**Cyt *b***
Paradise-crow	*Lycocorax pyrrhopterus*	NRM 569570	GQ334294	GQ334259	GQ334221
Glossy-mantled Manucode	*Manucodia ater*	NRM 566764	EU726210	EU726228	GQ334222
Jobi Manucode	*Manucodia jobiensis*	ZMUC100048	GQ334295	GQ334260	GQ334223
Curl-crested Manucode	*Manucodia comrii*	uncat.*			U15207
Crinkle-collared Manucode	*Manucodia chalybatus*	NRM 566755	GQ334296	GQ334261	GQ334224
Trumpet Manucode	*Phonygammus *(*Manucodia*)*keraudrenii*	NRM 566775	GQ334297	GQ334262	GQ334225
Long-tailed Paradigalla	*Paradigalla carunculata*	ZMUC100049	GQ334298	GQ334263	GQ334226
Short-tailed Paradigalla	*Paradigalla brevicauda*	NRM 566736	GQ334299	GQ334264	GQ334227
Arfak Astrapia	*Astrapia nigra*	NRM 551602	GQ334300	GQ334265	GQ334228
Splendid Astrapia	*Astrapia splendidissima*	NRM 569961	GQ334301	GQ334266	GQ334229
Stephanie's Astrapia	*Astrapia stephaniae*	NRM 551677	GQ334302	GQ334267	GQ334230
Ribbon-tailed Astrapia	*Astrapia mayeri*	AM O.45772	GQ334303	GQ334268	GQ334231
Huon Astrapia	*Astrapia rothschildi*	ZMUC 100463	GQ334304	GQ334269	GQ334232
Western Parotia	*Parotia sefilata*	NRM 561818	GQ334305	GQ334270	GQ334233
Carola's Parotia	*Parotia carolae*	NRM 566752	GQ334306	GQ334271	GQ334234
Lawes' Parotia	*Parotia lawesii*	NRM 569963	GQ334307	GQ334272	GQ334235
Eastern Parotia	*Parotia helenae*	ZMUC100047	GQ334308	GQ334273	GQ334236
Wahnes's Parotia	*Parotia wahnesi*	ZMUC 100462	GQ334309	GQ334274	GQ334237
King of Saxony Bird-of-Paradise	*Pteridophora alberti*	NRM 566740	GQ334310	GQ334275	GQ334238
Superb Bird-of-Paradise	*Lophorina superba*	NRM 566745	GQ334311	GQ334276	GQ334239
Paradise Riflebird	*Ptiloris paradiseus*	ZMUC 100062	GQ334312	GQ334277	GQ334240
Victoria's Riflebird	*Ptiloris victoriae*	ZMUC100043	GQ334313	GQ334278	GQ334241
Magnificent Riflebird	*Ptiloris magnificus*	NRM 569616	EU726211	EU726229	GQ334242
Growling Riflebird	*Ptiloris intercedens*	ZMUC100040	GQ334314	GQ334279	GQ334243
Black Sicklebill	*Epimachus fastuosus*	NRM 551601	GQ334315	GQ334280	GQ334244
Brown Sicklebill	*Epimachus meyeri*	NRM 569995	GQ334316	GQ334281	GQ334245
Buff-bailed Sicklebill	*Drepanornis *(*Epimachus*)*albertisi*	MV C148	EU380475	EU380436	U15205
Pale-billed Sicklebill	*Drepanornis *(*Epimachus) bruijnii*	ZMUC100045	GQ334317	GQ334282	GQ334246
Magnificent Bird-of-Paradise	*Diphyllodes (Cicinnurus*)*magnificus*	NRM 569677	GQ334318	GQ334283	GQ334247
Wilson's Bird-of-Paradise	*Diphyllodes *(*Cicinnurus*) *respublica*	NRM 566767	GQ334319	GQ334284	GQ334248
King Bird-of-Paradise	*Cicinnurus regius*	NRM 569661	GQ334320	GQ334285	GQ334249
Standardwing Bird-of-Paradise	*Semioptera wallacii*	ZMUC100061	GQ334321	GQ334286	GQ334250
Twelve-wired Bird-of-Paradise	*Seleucidis melanoleucus*	NRM 552057	GQ334322	GQ334287	GQ334251
Greater Bird-of-Paradise	*Paradisaea apoda*	ZMUC64493	GQ334323	GQ334288	GQ334252
Raggiana Bird-of-Paradise	*Paradisaea raggiana*	ZMUC100039	GQ334324	GQ334289	GQ334253
Lesser Bird-of-Paradise	*Paradisaea minor*	NRM 700230	GQ334325	GQ334290	GQ334254
Red Bird-of-Paradise	*Paradisaea rubra*	NRM 700233	GQ334326	GQ334291	GQ334255
Goldie's Bird-of-Paradise	*Paradisaea decora*	AM A.14473			GQ334256
Emperor Bird-of-Paradise	*Paradisaea guilielmi*	ZMUC100041	GQ334327	GQ334292	GQ334257
Blue Bird-of-Paradise	*Paradisaea rudolphi*	ZMUC100060	GQ334328	GQ334293	GQ334258
Short-tailed Batis/Fernando Po Batis	*Batis mixta/poensis*	MNHN CG 1998-783/ZMUC 02953	DQ406665	EU272120	DQ011862
Hooded Crow/Carrion Crow	*Corvus cornix/corone*	NRM 986167/MNHN CG 1995-41*	DQ406663	EU272116	AY228087
Spangled Drongo/Hair-crested Drongo	*Dicrurus bracteatus/hottentottus*	UBMW 68045/uncat.*	EF052813	EU272113	EF113121
Australian magpie	*Gymnorhina tibicen*	MV AC78/uncat.*	DQ406669	EU27119	AF197867
Black-faced Monarch	*Monarcha melanopsis*	MV B541	EU272089	EU272114	FJ821128
Tree Sparrow	*Passer montanus*	NRM 976359	AY336586	DQ785937	AY228073
Lovely Fairy-wren/Superb Fairy-wren	*Malurus amabalis/cyaneus*	MV C803/uncat.*	EF441219	EF441241	AF197845
Superb Lyrebird	*Menura novaehollandiae*	MV F722	EF441220	EF441242	AY064276
Yellow-bellied Elaenia	*Elaenia flavogaster*	NRM 966970	DQ435464	DQ435480	AF453807
Variable Antshrike	*Thamnophilus caerulescens*	NRM 967007	AY336587	DQ435504	AY078176
Velvet Asity	*Philepitta castanea*	ZMCU S458/FMNH 345690*	AY336591	DQ785938	AY065726
Rifleman	*Acanthisitta chloris*	NRM 569989	EU726202	EU726220	AY325307
Blue-fronted Amazon/Maroon-bellied Parakeet	Amazona aestiva/Pyrrhura frontalis (Psittacidae)	NRM 966989, uncat*	AY194432	DQ881775	AY751643

Acronyms: NRM = Swedish Museum of Natural History, Stockholm; ZMUC = Zoological Museum of the University of Copenhagen. All samples excect those marked with an asterisk are from vouchered specimens.

For extractions, amplifications, and sequencing procedures from study skin samples we followed the procedures described in Irestedt *et al*. [28]. Several new primers were also designed (Table 2).

**Table 2:** Primers designed for this study.

**Primer name**	**Primer sequence (5' - 3')**	**Region**
Cytb-BopF1	TCA CAC AAA TTA TCA CAG GCC T	Cytochrome b
Cytb-BopF2	TCC TCC TAA CCC TAA TAG CAA C	Cytochrome b
Cytb-BopF3	CCT ACA CGA AAC AGG ATC AAA CAA	Cytochrome b
Cytb-BopF4	CTC CCC ATA TCA AAC CAG AAT GAT A	Cytochrome b
Cytb-BopR1	TCC GAC GAA GGC TGT TGC TAT TA	Cytochrome b
Cytb-BopR2	GGG GGT TGT TTG ATC CTG TTT C	Cytochrome b
Cytb-BopR3	TCG GAG GAT GGC GTA TGC AAA TAG	Cytochrome b
Cytb-BopR4	AAT GGA TGT TCG ACT GGT TGG CT	Cytochrome b
G3P-BopintF1	AAT CCC ACT GTG GAG TGA GAT TGT	GAPDH intron 11
G3P-BopintR1	AGG AGG CAG CTA CAG TAA TTT CAG GT	GAPDH intron 11
ODC-Bop-F2	CAG ACC CAG AGA CCT TTG TTC A	ODC intron 6 and 7
ODC-Bop-F3	GTA GCT TAC TTT GAC CAG CTT GGC A	ODC intron 6 and 7
ODC-Bop-R1	AGT TGC CAA TTT TAG TGC ATC AGT	ODC intron 6 and 7
ODC-Bop-R3	AAA CAG AGG TAA CTC ATG TTC AAG T	ODC intron 6 and 7

The primers have been designed to amplify short regions (~200-300 bp) from degraded DNA obtained from museum study skin samples.

### Phylogenetic analyses and estimation of speciation rates

We used Bayesian inference (see e.g., [29]) to estimate phylogenetic relationships. The models for nucleotide substitution used in the analyses were selected for each gene individually by applying the Akaike Information Criterion (AIC, Akaike [30]) using the program MrModeltest 2.2 [31] in conjunction with PAUP* [32]. Due to a rather low number of insertions in the non-coding nuclear loci the sequences could easily be aligned by eye. All gaps were treated as missing data in the analyses.

Posterior probabilities of trees and parameters in the substitution models were approximated with MCMC and Metropolis coupling using the program MrBayes 3.1.1 [33]. Analyses were performed for the cytochrome *b *gene, a concatenated data set of the nuclear loci (GAPDH and ODC), and a concatenated data set of all genes. In the analyses of the concatenated data sets the models selected for the individual gene partitions were used. We used an unconstrained, exponential branch length prior. All chains were run for 25 million generations, with trees sampled every 100 generations. The trees sampled during the burn-in (i.e., before the chain had reached its apparent target distribution) were discarded, and after checking for convergence, final inference was made from the concatenated output from the two runs.

Sexual selection has been considered a driving force behind speciation [22-25]. By comparing speciation rates between clades with and without sexual selection it could be possible to investigate if sexual selection promotes speciation (e.g., [34-36]). The temporal variation in diversification rates is reflected by the variation in branch lengths in a chronogram and can be used to estimate the overall speciation rates as well as relative contribution of extinction [37]. However, this requires taxonomically almost complete chronograms which is still lacking for the core Corvoidea. However, by using the methods described by Magallon and Sanderson [38] it is possible to estimate the diversification rate and the corresponding 95% confidence interval for the entire core Corvoidea, and compare this with the diversification rate of the polygynous birds-of-paradise. As the confidence interval will depend on the relative extinction rate (which is unknown) we calculated confidence intervals for both a high (ε = 0.90), and a zero (ε = 0) relative extinction rate. By using previously published phylogenies and divergence time estimates [39-41] it is possible to roughly estimate the divergence rates for Dicruridae and Monarchidae, two bird families closely related to the birds-of-paradise within the core Corvoidea assemblage. As the available divergence time estimates for these groups are based on other calibration points that suggest slightly different ages on comparable nodes, or only show relative age estimates, these estimates have been rescaled to be consistent with our chronogram. The rescaling was done by calculating the relative age between the two families, respectively, and the *Corvus *node (a node present in all trees) and multiply this age with our age estimate of the corresponding split. We consequently used the method to calculate diversification rates for crown groups [38].

### Estimation of birds-of-paradise divergence times

We used a relaxed clock model implemented in Beast 1.4.7 [42-44] to estimate divergence times between phylogenetic lineages based on the concatenated dataset of all genes. To calibrate the tree we used the geological split between New Zealand and Antarctica, as it has been associated with the basal separation of the *Acanthisitta*-lineage from all other passerines [45,46]. The dating of this split has often been assumed to be around 85-82 Mya [47,48], but more recently the timing of this split has been suggested to be more uncertain, 85-65 Mya [49,50]. In order to account for this uncertainty we used a normal distributed tree prior with a median at 76 Mya and a standard deviation of 8 (quintiles 2.5% = 60.3 Mya, 5% = 62.8 Mya, 95% = 89,2 Mya, 97.5% = 91.7 Mya). As for other priors, we used all default settings, except for the Tree Prior category that was set to Yule Process and an uncorrelated lognormal distribution for the molecular clock model. We used a GTR+Γ model and ran MCMC chains for 25 million generations.

## Results

### Variation in the molecular data set, model selection

For all taxa 841 bp of the cytochrome *b *gene was sequenced. As we used partially overlapping primers the region covers a 868 bp region (ending 125 bp from the 3' end in the cytochrome *b *gene). Taking into account that for some taxa a few short fragments are missing, the alignments of the non-coding intron regions are 387 bp for GAPDH (the individual sequences ranged between 247-269 bp), and 685 bp for ODC (ranging between 577-619 bp for all taxa, except *Corvus *that, due to a large deletion, is only 429 bp). Some indels in more variable regions were found to be autapomorphic, while most other indels are congruent with the phylogenetic tree obtained from the analysis of the combined data set. The only exceptions are a 4 bp deletion shared between all *Paradisaea *and *Monarcha *and a 1 bp insertion shared by the *Parotia*-*Pteridophora *clade and some outgroup taxa in the GAPDH locus.

**Figure 1:**
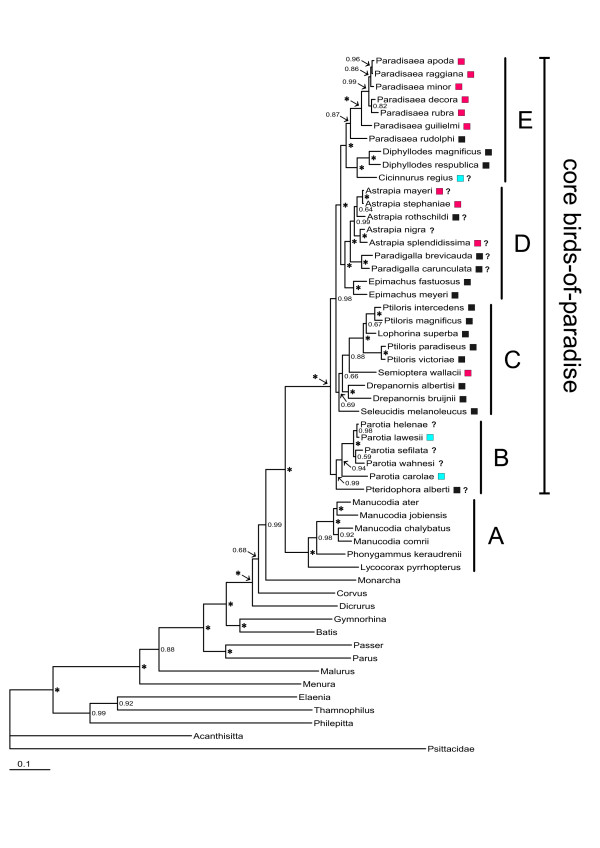
**Phylogenetic relationships of the birds-of-paradise**. The 50% majority rule consensus tree obtained from the analyses of the combined data set (cytochrome *b*, ornithine decarboxylase introns 6 and 7, and glyceraldehyde-3-phosphate dehydrogenase intron 11). Posterior probability values are indicated above the nodes, posterior probability values of 1.00 are indicated with an asterisk. Male display strategies in core birds-of-paradise according to (Frith and Behler 1998) are indicated by boxes to the right of the taxon names (red = lek, blue = exploded lek, black = solitary). Question marks to the right of the boxes indicate that display strategy is not fully established.

The prior selection of substitution models supported the GTR+I+Γ model for cytochrome *b*, the HKY+ Γ for GAPDH, and GTR+Γ for ODC. After discarding the burn-in phase the final inference was based on a total of 200 000-225 000 samples from the posterior for all conducted Bayesian analyses. For the phylogenetic inference of the concatenated data set of all genes, the mode of the posterior distribution of topologies is presented as a 50% majority-rule consensus tree (Figure 1).

### Phylogenetic relationships

The trees obtained from the analyses of cytochrome *b *and the nuclear data sets receive less support than the tree obtained from the analysis of the concatenated data set. Nevertheless, several clades are recognized in all analyses, and conflicts exclusively concern weakly supported nodes (posterior probabilities < 0.95). Most deviant in relation to the combined tree is the sister relationship between *Epimachus *and *Drepanornis *(pp = 0.83) suggested only by the cytochrome *b *tree. In the cytochrome *b *tree *Monarcha *is also nested between the *Manucodia*, *Phonygammus *and *Lycocorax *clade and core birds-of-paradise (pp = 0.90).

Most nodes in our combined phylogeny receive strong support (pp > 0.95) and are often recovered by both the nuclear and the mitochondrial genes. Thus, we consider our combined phylogeny a reliable estimate of the evolutionary relationships among the birds-of-paradise.

Five main clades are recognized in our phylogeny (A-E in Figure 1). The first clade (A) consists of manucodes (*Manucodia *and *Phonygammus*) and the paradise crow (*Lycocorax pyrrhopterus*). This clade is supported as the sister group to the core birds-of-paradise (remaining genera). The king of Saxony bird-of-paradise (*Pteridophora alberti*) and parotias (*Parotia*) form the second clade (B). Within this clade *P. carolae *is highly divergent and warrants separate generic treatment.

The third clade (C) consists of the twelve-wired bird-of-paradise (*Seleucidis melanoleuca*), *Drepanornis *sicklebills, the standardwing bird-of-paradise (*Semioptera wallacii*), riflebirds (*Ptiloris*), and the superb bird-of-paradise (*Lophorina superba*). However, support for more basal nodes within this clade is low, and the affinity of the twelve-wired bird-of-paradise, *Drepanornis *sicklebills, and the standardwing bird-of-paradise should be regarded as provisional. Although support values are low, New Guinean *Ptiloris *and *Lophorina *form a clade separate from the Australian *Ptiloris*. This apparent relationship is worth investigating further as it suggests a significant phylogenetic break between Australia and New Guinea.

The fourth clade (D) includes sicklebills (*Epimachus*), paradigallas (*Paradigalla*) and astrapias (*Astrapia*). The fifth clade (E), consists of *Paradisaea *birds-of-paradise, Wilson's bird-of-paradise (*Diphyllodes respublica*), magnificent bird-of-paradise (*Diphyllodes magnificus*), and king bird-of-paradise (*Cicinnurus regius*). Within this clade the genera *Diphyllodes *and *Cicinnurus *are sister lineages, while the blue bird-of-paradise (*Paradisaea rudolphi*) is sister to other *Paradisaea *birds-of-paradise.

### Divergence time estimates and speciation rates

**Figure 2:**
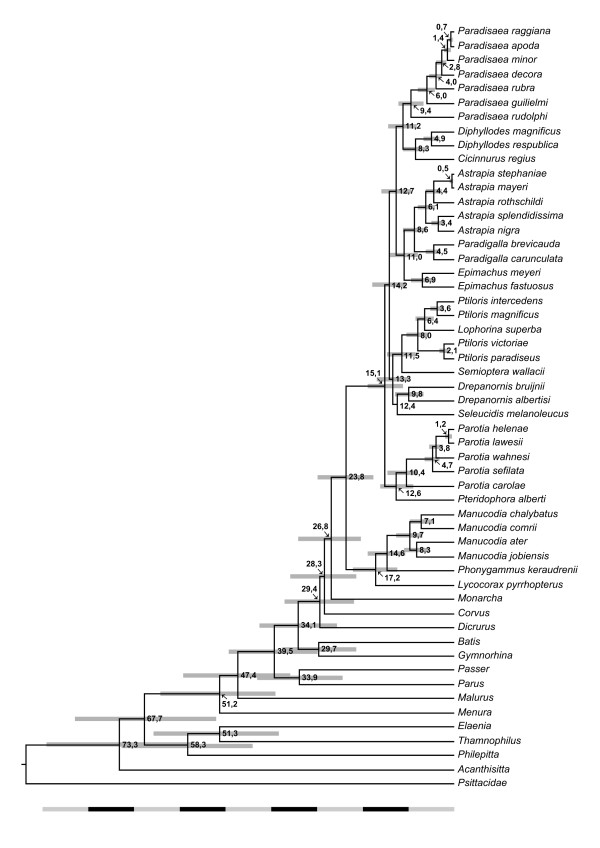
**Chronogram with divergence times estimates of the birds-of-paradise**. The divergence times and confidence intervals (grey bars) were estimated under a relaxed clock model implemented in Beast 1.4.7 [33]. For the calibration of the chronogram the postulated separation of *Acanthisitta *from all other passerines in the phylogeny was used.

All nodes supported by posterior probabilities above 0.95 in the tree obtained from the Bayesian analyses of the concatenated data set of all genes (Figure 1) are recognized in the chronogram. The chronogram and divergence time estimates are shown in Figure 2. The split between the *Manucodia*/*Lycocorax *clade and core birds-of-paradise clade is estimated to have occurred around 24 million years ago, while the basal divergence of the polygynous core birds-of-paradise is suggested to be around 15 million years old. The age estimates further suggest that generic separations occurred between 6 and14 million years ago, while speciation within genera mostly occurred between 0.5 and 10 million years ago.

The calculated diversification rates and the 95% confidence intervals based on the mean diversification rate for the core Corvoidea at a relative extinction rates of ε = 0 and ε = 0.90 are shown in Table 3 (expressed as expected number of species). The results suggest that the speciation rates within the entire birds-of-paradise clade as well as within the restricted polygynous core birds-of-paradise fall within the 95% confidence intervals for the core Corvoidea at both low (ε = 0) and high (ε = 0.90) relative extinction rates.

**Table 3:** Rates of diversification.

**Clade**	**Nummer of species**	**Age**	**Diversification rates (ε = 0)**	**Diversification rates (ε = 0.90)**	**Expected number of species (ε = 0)**	**Expected number of species (ε = 0.90)**
Birds-of-paradise	40	23.8	0,13	0,06	2-131	4-455
core birds-of-paradise	34	15.1	0,19	0,09	1-35	2-156
Monarchidae	~130	~10.6	~0,39	~0,24	~1-17	~1-83
Dicuridae	~24	~20.0	~0,12	~0,06	~2-74	~3-290
core Corvoidae	755	39.5	0,15	0,11	11-1402	19-2658

Absolute rate of diversification for the entire birds-of-paradise clade, core (polygynous) birds-of-paradise, some closely related families, and core Corvoidea, estimated in absence of extinction (ε = 0) and comparatively high (ε = 0.90) relative extinction rate. The 95% confidence intervals at relative extinction rates of ε = 0 and ε = 0.90 are expressed as expected number of species for each clade. The 95% confidence intervals have been calculated as described by Magallon and Sanderson [Magallon and Sanderson 2001] and is based mean diversification rate for the core Corvoidea (at a relative extinction rates of ε = 0 and ε = 0.90) and the age of the clades.

## Discussion

### Evolution of sexual dimorphism

**Figure 3:**
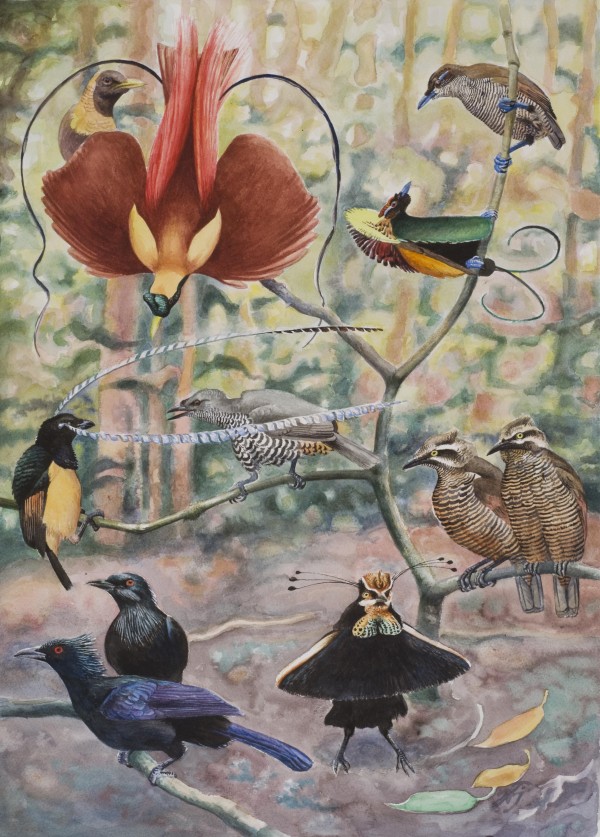
**Examples of plumage diversity and sexual dimorphism in birds-of-paradise**. Lower left male and female of the monogamous *Manucodia keraudrenii*, lower right male and two females of *Parotia carolae*, center left male and female *Pteridophora alberti*, top left male and female *Paradisaea rubra*, and top right male and female *Diphyllodes magnificus*.

Our divergence time estimates suggest that the birds-of-paradise originated approximately 24 million years ago, which renders the family older than previously suggested by DNA-DNA hybridization data [10] and allozyme data [9,11]. Particularly noticeable is the old age (ca 15 My) of the sexually dimorphic and polygynous core birds-of-paradise. Although the variation in male plumage ornamentations is astonishing within the core birds-of-paradise (Figure 3), most of this variation is found between genera that diverged around 10 million years ago. More interesting is that morphologically homogeneous genera seem to be quite old. One of the most prominent examples is the genus *Paradisaea*. In this genus all lekking species are morphologically very homogeneous although the age of the genus is found to be more than 6 million years old (the split between *P. guilielmi *and other lekking *Paradisaea *species). Similarly, the morphological variation is modest within the genus *Parotia*, for which the exploded lekking system is estimated to be around 10 million years old. Other sexually dimorphic and polygynous genera such as *Ptiloris *and *Astrapia *also show little morphological diversification between species. The calculated diversification rates (Table 3) further indicate that the speciation rate in core birds-of-paradise is not excessively high. In fact the speciation rate seems to be more similar to the speciation rate found for the sexually monomorphic drongos (Dicruridae) than to the speciation rate found for the sexually dimorphic monarch flycatchers (Monarchidae).

Christidis and Schodde [51] postulated that the evolution of spectacular variation in male plumages in birds-of-paradise could be explained by female sexual selection for novel partners (but see [52,53]). This model was proposed to explain the apparent rapid radiation of the birds-of-paradise. However, in the present study we find no evidence that sexual selection has promoted a particularly rapid morphological diversification [19,20] or an exceptionally high rate of speciation [22-25] in birds-of-paradise.

Among passerine birds there is probably no other family with so many reported cases of interspecific and intergeneric hybrids in the wild as in the birds-of-paradise [1]. For some genera where several species tend to have interconnected distributions, such as *Paradisaea*, gene flow through hybridization may have constrained speciation and phenotypic diversification. However, in most genera within core birds-of-paradise, species and subspecies are geographically well separated (i.e. *Astrapia *and *Parotia*), and modest speciation and phenotypic differentiation rates are thus difficult to explain by hybridization. Although little is known about the age when birds-of-paradise breed for the fist time, it is assumed that males of polygynous species do not reproduce until they have obtained full adult plumages, which happens several years later than in females [1]. A longer generation time in polygynous male birds-of-paradise, compared to other passerine birds, may thus influence the inheritance rate of phenotypic characters in polygynous species of birds-of-paradise. Ecological factors, such as abundant food sources in New Guinea, may also explain that male birds-of-paradise have been able to develop and maintain promiscuous breeding, magnificent plumages and elaborate courtship displays.

Birds-of-paradise are among the few bird groups that have developed a social system based on promiscuous mating and arenas or leks [54]. It is therefore notable that our phylogeny suggests that the degree of lekking behaviour (from large communal lekking to solitary display) varies considerably within the polygynous core birds-of-paradise, showing no clear phylogenetic structure (Figure 1). It is also notable that the strong sexual dimorphism within core birds-of-paradise has been lost in the genus *Paradigalla*.

### Sexual behaviour and dispersal ability

Groups closely related to the birds-of-paradise, such as Monarchidae, Rhipiduridae and Dicruridae [41,55-57] have all dispersed to other continents and remote oceanic islands. The birds-of-paradise on the other hand have only colonized islands within the New Guinea orogen, except two species (*Lycocorax pyrrhopterus *and *Semioptera wallacii*) inhabiting Halmahera and surrounding islands of the North Moluccas. The present distributions can largely be explained by vicariance, considering the fluctuating sea levels in the Late Miocene and Pliocene [58]. This raises the question if the development of the special reproductive behaviour of birds-of-paradise may have constrained the capacity to disperse.

In four genera of birds-of-paradise the sexes are morphologically similar: *Manucodia*, *Phonygammus*, *Paradigalla *and *Lycocorax*. These species are monogamous and form pair bonds, which is quite unlike the promiscuous behaviour of the rest of the family. This less "spectacular" life strategy probably enabled these birds to have a higher dispersal capacity and could explain the present day occurrence of *Manucodia *and *Phonygammus *on several islands off the New Guinea coast and on Australia's Cape York Peninsula, and of *Lycocorax *on the North Moluccas. *Paradigalla *on the other hand is a genus restricted to the central New Guinean mountain range, which renders it less likely to disperse. The present day distribution of *Semioptera wallacii *in the North Moluccas is more difficult to explain. However, molecular clock estimates (Figure 2) suggest that it diverged from other birds-of-paradise in the late Miocene at a time when these islands, which are of oceanic origin, drifted close by the Vogelkop peninsula [59,60].

We propose that the main reason why the core birds-of-paradise, unlike other corvoid bird families, have only diversified within New Guinea and islands in the immediate vicinity is linked with their promiscuous breeding system. It is likely that the costs not only involve handicaps associated with ornamental plumes and intensive displaying, but also involve a strong attachment to display sites [1]. Furthermore, the fact that males and females live separately, except during mating time, means that successful long-distance dispersal and establishment of new breeding populations are less likely.

A similar sedentary life strategy, with limited dispersal outside mainland areas (and islands which have been connected with these areas) is evident in several other families with elaborate plumages, such as Phasianidae [61], Pipridae and the polygynous species within Cotingidae [62].

### Patterns of speciation and diversification

Within several lineages of birds-of-paradise (*Astrapia*, *Ptiloris *and *Paradigalla*) there are distinct allopatric clades distributed in the east and the west of New Guinea, which separated about 3 - 6 million years ago (Figure 2). Allopatric speciation also appears to be the major mode of diversification within *Paradisaea*, which is a mainland lowland species complex that originated in the Pleistocene, while island species (*P. rubra *and *P. decora*) are slightly older. However, the divergences involving the mountain species (*P. guilielmi *and the highly divergent *P. rudolphi*) are much older. Within *Ptiloris*, the time separation of New Guinean and Australian taxa corresponds to broad land connections in the upper Miocene and marine transgression in the Pliocene [59].

Heads [63-65] has argued that present day distributions of birds-of-paradise in New Guinea are difficult to explain simply by Pleistocene refugia processes, and rather that biogeographical patterns should be seen in light of historic terrane movements over a longer time span. As support for this hypothesis he used three birds-of-paradise genera (*Astrapia*, *Parotia*, and *Paradisaea*) for which assumed sister relationships suggest a strong biogeographical connection between the Vogelkop and the Huon Peninsula in western and eastern New Guinea, respectively. Our phylogeny does not provide support for this biogeographical scenario. A sister relationship between *Parotia sefilata *in the Vogelkop and *Parotia wahnesi *on the Huon Peninsula is only weakly supported (PP = 0.59), and within the genus *Astrapia *our phylogeny strongly suggests sister relationships between species that occur in closely connected geographical areas (between Huon Peninsula and the Central Highlands, and between the Vogelkop and the Star Mountains). Within the genus *Paradisaea*, *P. guiliemi *from the Huon Peninsula is sister to all other *Paradisaea *species (except *P. rudolphi*, a morphologically rather divergent montane species) occupying almost all lowland areas in New Guinea.

The two species of *Drepanornis *as well as the two species of *Epimachus *separated about 10 and 7 million years ago, respectively. While the two species of *Drepanornis *occupy different elevations in low- and mid-montane forests, the two species of *Epimachus *are altitudinal replacements in mountain forests. Consequently, these two cases could represent old cases of altitudinal speciation. *Parotia lawesii *and *Parotia helenae *have similar patchy distributions as *Epimachus *across the mountains of New Guinea, and may represent a recent altitudinal speciation event. Examples of presumed altitudinal speciation in New Guinea have also been reported in *Meliphagidae *honeyeaters [66].

## Conclusion

The phylogenetic results suggest that the birds-of-paradise could be subdivided into five main clades (A-E in Figure 1). Some of these clades confirm traditionally recognized relationships while others are novel hypotheses. The divergence time estimates further suggest that birds-of-paradise constitute an older clade than previously suggested. It appears that diversification within several genera of birds-of-paradise has been a continuous process through the Tertiary and that younger divergences are geographically rather closely linked. In addition to allopatric speciation, there appear to be some examples of altitudinal speciation. Particularly interesting is the observation that sexually dimorphic polygynous genera are morphologically homogeneous although divergences between species are suggested to be several million years old, and that calculations of diversification rates indicate that the speciation rate is not excessively high. Thus, sexual selection in birds-of-paradise appears not to have generated a particularly rapid change in sexual ornamentations or a markedly high speciation rate. Although the explanation remains uncertain, a long generation time in polygynous male birds-of-paradise and extensive hybridization may have constrained morphological diversifications and speciation. External ecological factors, such as abundant food sources in New Guinea, may also explain why male birds of paradise have been able to develop and maintain promiscuous breeding systems, magnificent plumages and elaborate courtship displays.

## Authors' contributions

MI designed the study, carried out the labwork, performed the phylogenetic analyses, and drafted the manuscript. PE and KJ assisted with the phylogenetic analyses and conceived the study. JF, KJ and LC assisted with the design of the study and with the draft of the manuscript. All authors read and approved the manuscript.
